# Integrated CAD/CAM Approach for Parametric Design and High Precision Fabrication of Planar Curvilinear Structures

**DOI:** 10.3390/mi16070805

**Published:** 2025-07-11

**Authors:** Jonas T. Churchill-Baird, O. Remus Tutunea-Fatan, Evgueni V. Bordatchev

**Affiliations:** 1 Mechanical and Materials Engineering, Western University, London, ON N6A 5B9, Canada; 2 Automotive and Surface Transportation Research Centre, National Research Council of Canada, London, ON N6G 4X8, Canada

**Keywords:** curvilinear V-grooves, integrated CAD/CAM framework, single point diamond cutting (SPDC), five-axis machining, design and fabrication of functional surfaces

## Abstract

Curvilinear V-grooves are increasingly employed in functional surfaces with applications ranging from fluidics to tribology and optics. Despite their widespread use, the accurate and repeatable fabrication of curvilinear V-grooves remains challenging due to their inherent geometric complexity and the lack of relevant commercial CAD/CAM systems. To address this, the present study proposes a CAD/CAM integrated framework capable of automating the design and fabrication of functional surfaces comprising curvilinear V-grooves generated by multi-axis single-point diamond cutting (SPDC). The framework is organized into three main functional blocks supported by seven secondary modules that encompass the entire process from V-groove geometry definition to cutting. The developed framework was practically validated by fabricating sinusoidal V-grooves on a flat surface and testing the capillary flow functionality of a curvilinear pattern. These results demonstrate the relevance of the integrated framework to curvilinear V-groove fabrication, thereby offering a versatile solution for certain types of surface engineering applications.

## 1. Introduction

Many new and improved functional parts and tooling surfaces have been endowed with augmented functionality through the use of high-precision microfabrication technologies. Advancements in the modelling of functional micro/nano-scale geometric structures have been shown to provide surfaces with augmented and/or unique functionalities for various applications such as micro-fluidics [1], friction control [2], light-guiding and trapping [3], and aero- and hydrodynamics [4,5].

Among the most common micro-/nano-scale geometric structures, V-grooves have emerged as versatile elements that enable enhanced surface functionality across a range of fields. In microfluidics, they support capillary flow in both open and closed channels [6,7], enable the rapid transport of fluids such as water and ink [1], and facilitate efficient thermal management in high-heat-flux applications [8]. Vertical V-groove networks have demonstrated gravity-defying capillary rise [9], and ink-filled V-grooves have been used to fabricate inexpensive microelectrical circuits [10]. In tribological applications, they improve lubricant flow and surface performance during forming processes [2]. V-grooves have also been used in optical applications, such as improving photovoltaic efficiency [11] and creating light guides with high-illumination efficiency [3]. Additionally, bioinspired V-groove arrays have been developed for anisotropic droplet transport and passive fluid manipulation [12]. These diverse applications underscore the growing demand for the automated, high-precision fabrication of curvilinear V-groove structures. This work addresses the current lack of CAD/CAM tools that enable the efficient modeling, path generation, and micromachining of such features on planar substrates.

Given the numerous industrial applications of V-grooves, their fabrication tends to be an important obstacle, particularly when the targeted geometry involves curvilinear or freeform paths that require precise control of the tool orientation, cutting forces, and surface quality. Nonetheless, the high-precision and cost-efficient manufacturing of ultraprecise micro-/nano-scale structures is far from being a trivial task. More specifically, a high surface quality of the groove facets (Sa < 10 nm) coupled with burr-less structures is of utmost importance [13] because the achievement of these qualities provides the most accurate and optimized functional surfaces. In addition to the more conventional five-axis micromachining [14], multi-axis single-point diamond cutting (SPDC) is one of the most effective microfabrication machining techniques. SPDC has already been used for the fabrication of micro-V-grooves on non-ferrous materials [15], with advanced SPDC being employed with six-axis motion control to produce highly precise and accurate micro-V-grooves [16]. With this in mind, the V-grooves created using SPDC are an “inverse replica” of the cutting tool geometry. Several different strategies can be implemented with SPDC to avoid breakage and bending of the tool and/or high-aspect-ratio riblets [17]. Available strategy implementations include those relying on a constant chip thickness or a constant cutting area/force, which can be executed using single flank, alternate flank, and/or double flank cutting [17]. While other microfabrication methods, such as laser irradiation [3,18] and multiphoton polymer structuring [19], can produce microscale geometries, they typically lack the geometric fidelity, surface quality, or material flexibility required for high-precision curvilinear groove machining. In contrast, SPDC offers deterministic control over tool geometry and motion, making it ideally suited for fabricating continuous, curved V-groove features with sub-micron accuracy. However, the lack of automated CAD/CAM tools for modeling and generating toolpaths for such structures remains a critical gap, particularly when complex trajectories or varying tool orientations are involved.

To this end, the lack of CAD/CAM solutions that can automate the modelling and post-processing of a tool path trajectory for micro-cutting V-grooves constitutes the foundation for the present study. These developments facilitate the creation of intricate microfluidic channels and other functional surfaces with enhanced capabilities. The present work extends our previous studies to include curvilinear V-groove-based functional microstructures that have not been addressed in the past. This need is amplified by the growing interest in optimized toolpath strategies for machining complex freeform surfaces, where conventional approaches may fail to account for local curvature or tool orientation constraints [20]. Innovative toolpath planning methods have been developed to address the challenges of multi-axis machining of complex geometries [21]. In most situations, these approaches optimize the tool orientation and movement, ensuring the accurate fabrication of curved microstructures.

To address the relative paucity of commercial systems used during the SPDC-based fabrication of curvilinear grooves, the current study focused on the development and advancement of a CAD/CAM integrated approach that can automate the solid modelling as well as the CAM post-processing of V-groove-based functional structures. The proposed framework includes several secondary functional blocks (SFB): (i) parametric model definition of a rectangular workpiece, (ii) parametric model definition of the V-groove cross-section and the defined curve, (iii) parametric model definition of a flat planar functional surface as an array of specifically placed curvilinear V-grooves, (iv) parameters of the cutting tool and its geometry, (v) discretization of the curvilinear pattern, (vi) cutting strategy of a unit V-groove, and (vii) cutting process plan. These seven SFBs comprise three main function blocks (MFB): (I) parametric model of the functional structure, (II) CAD module, and (III) CAM module. MFBs integrate the automated generation of a solid model and the tool path trajectory of V-groove-based functional structures. Particular effort was placed on the automated generation of a set of curvilinear V-grooves on a flat planar surface and their precise micromachining by multi-axis single-point diamond cutting (SPDC) supported by the rotation tool center point (RTCP) function [22] and chordal deviation. The proposed framework can be extended and used for various combinations of a single and/or a set of spatially oriented curvilinear V-grooves that can be applied to many other types of applications similar to those mentioned above.

## 2. Integrated CAD/CAM Approach

Developing a new and efficient functional structure can be difficult and time-consuming because such structures require interlaced considerations of the geometrical design, required or optimized functionality, and high-precision and cost-effective manufacturability. These considerations are the main drivers for an integrated approach based on several steps that link parameterization with modelling and microfabrication of the structures that can be added to the product surface to provide new or enhance existing functionality. Implementing the approach will provide a new or improved structure that can be rigorously tested to evaluate the physical performance and can be further improved to enhance the functionality, if necessary.

**Figure 1:**
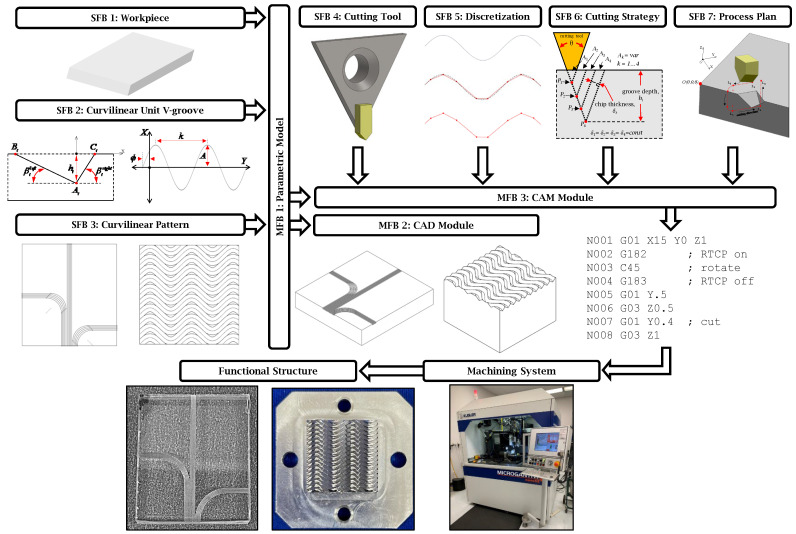
Block diagram of the proposed integrated CAD/CAM approach for parametric design and high-precision microfabrication of planar functional structures using curvilinear V-grooves.

Therefore, the focus of the developed framework presented in Figure 1 is on fully automating the process of the parametric design of curvilinear V-grooves and their high-precision fabrication on planar functional surfaces. The CAD/CAM integrated approach automates three main steps with functional blocks related to: (i) parameterization of the geometry of the workpiece and the unit V-groove as a defined curve, (ii) functional structure as repeated defined curves, or an inputted array of separate undefinable curves across the surface, and (iii) discretization of the patterns into a tool path trajectory, cutting tool geometry and its stance, strategy for cutting a V-groove, and process plan for cost-efficient and high-precision microfabrication of the entire functional structure. Each of these functional blocks (FBs) depicts a specific operation or a set of geometric parameters that define a particular design component. The two intended outputs of the approach are obtained through the links between the FBs, which present the flow of information. The two outputs are as follows: (a) a CAD solid model of the functional structure for its visualization and further numerical simulation of its functional performance, and (b) an NC code/program for micromachining of the structure.

Figure 1 presents an integrated approach that was divided into three main functional blocks (MFB): Parametric Module (MFB 1), CAD Module (MFB 2), and CAM Module (MFB 3). These MFBs take inputs from several secondary functional blocks (SFBs), termed as Workpiece (SFB 1), Curvilinear Unit V-groove (SFB 2), and Curvilinear Pattern (SFB 3). These three SFBs define the parameters that serve as inputs to MFB 1. The output of MFB 1 is a set of mathematical formulas that make up the parametric geometric model of the functional structure to integrate the use of MFB 2 and MFB 3. MFB 2 produced a solid model of the functional structure for further visualization and numerical simulation of its functional performance. MFB 3 additionally takes inputs from SFB 4–7, termed as Cutting Tool (SFB 4), Discretization (SFB 5), Cutting Strategy (SFB 6), and Process Plan (SFB 7), and functions as a post-processor to generate NC code to be used to fabricate an entire functional surface in accordance with the selected parameters of the cutting tool, cutting strategy, and process plan. The fabricated surface is a physical replica of a digital solid model. The surface is created by passing the generated NC code to a micromachining system (Figure 1). The performance of the fabricated surface can then be experimentally tested in order to verify/validate its newly acquired functionality.

## 3. Parametrization of the Workpiece, Curvilinear Unit V-Groove, and Curvilinear Pattern Geometries

The first main function block (MFB 1) is labeled as the parametric model in Figure 1 of the integrated CAD/CAM approach. Three geometric components of the functional structure (workpiece, curvilinear unit V-groove, and curvilinear pattern) are defined in SFBs 1–3 and are the inputs to MFB 1 (Figure 1). These components, with the exception of the input pattern option of SFB 3, are all linked by underlying trigonometric equations.

### 3.1. Workpiece Geometry (SFB 1)

**Figure 2:**
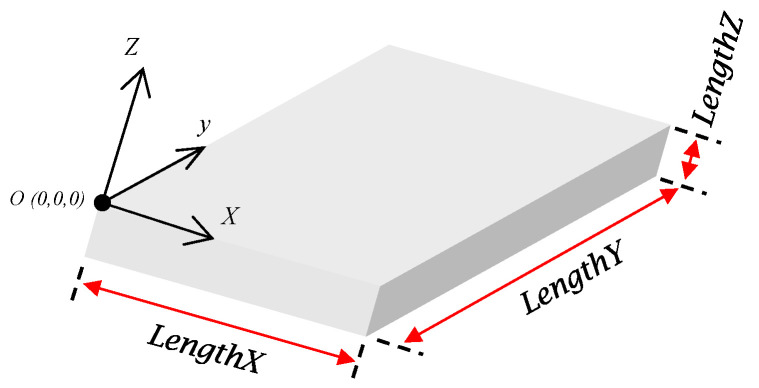
Parameterization of workpiece geometry.

This secondary functional block represents the first step in parameterizing functional structures. The location of the origin and the definition of the workpiece coordinate system (WCS) are the main aspects of SFB 1. These aspects are used to define three important inputs: the length of the workpiece in the X, Y, and Z directions in terms of the WCS, labeled as *LengthX*, *LengthY*, and *LengthZ*, as shown in Figure 2.

The size of the working area of the workpiece is defined using these three inputs and can be further used to define the parametric model. This first step is the foundation stone for the trigonometric equations used to define the other two geometric components. A basic rectangular stock was considered as the logical start for the proposed approach and will be significantly expanded further to include more complex surfaces, including free-form geometries.

### 3.2. Curvilinear Unit V-Groove (SFB 2)

**Figure 3:**
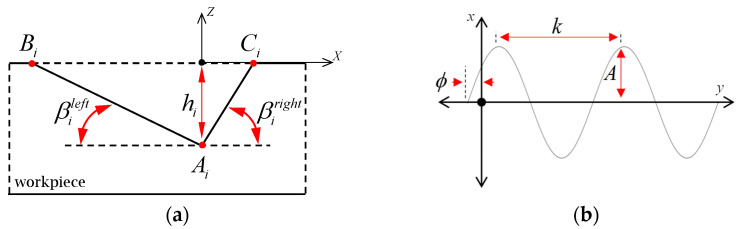
Parameterization of the unit V-groove: (**a**) cross-sectional geometry and (**b**) sample of the sweeping geometry.

This particular secondary block combines two features of the unit V-groove. The first feature, as shown in Figure 3a, is the cross-section of the “curvilinear” V-groove geometry, which is defined by the shape of the cutting tool. The parameterization of this cross-section has only one constant, which is the triangular shape, as the grooves are “inverse replicas” of the triangular diamond cutting tool. Under this condition, no spatial movements or alignments of the cutting tool are required, which significantly simplifies the microfabrication process. The SFB 2 allows for the customization of each individual groove to the *i*-th groove orientation angle. Depending on the number of grooves, there is a set of *i* = 1…*n* V-grooves, where each *i*-th groove cross-section can be set differently from the last groove.

Figure 3a presents the parameterization of the V-groove geometry that is generally defined by its triangular cross-section $\left\{A_{i}\left(x,y,z\right),{\;B}_{i}(x,y,z),{\;C}_{i}(x,y,z)\right\}$ to the *i*-th groove angle. The first of five input parameters is the groove depth, $h_{i}$, that is the *Z* coordinate of the apex point $A_{i}\left(z\right)$ and located vertically below the origin point for simplification, and then is redetermined relative to the origin in SFB 3 per the *i*-th groove. The next two inputs are the left and right facet angles, $\beta_{i}^{\mathrm{left}}$ and $\beta_{i}^{\mathrm{right}}$, which determine the overall apex angle of the V-groove cross-section. These three inputs, $h_{i}$, $\beta_{i}^{\mathrm{left}}$, $\beta_{i}^{\mathrm{right}}$ define the triangular cross-section of each *i*-th V-groove $\left\{A_{i},{\;B}_{i},\;{\;C}_{i}\right\}$.

The next feature of this SFB is the choice to define a parametric or explicit equation of a curve. In defining a curve, the choice is made to have a predefined pattern that can start from one point to another and can subsequently be repeated if desired. This curve driving the sweeping feature/pattern can be expressed in an explicit form. A typical example in this direction is depicted in Figure 3b, which shows a sinusoidal sweeping pattern characterized by the amplitude *A*, period *k*, and phase *ϕ*.

This curve can be used to guide the cross-section shown in Figure 3a, and therefore create a curvilinear feature. More specifically, once the parametric equation of the driving curve is provided to SFB 2, the end and start points of the unit V-groove can be defined as $t_{i}^{\mathrm{start}}$ and $t_{i}^{\mathrm{end}}$. This approach can be virtually extended to any parameter-defined curve.

### 3.3. Curvilinear Pattern (SFB 3)

**Figure 4:**
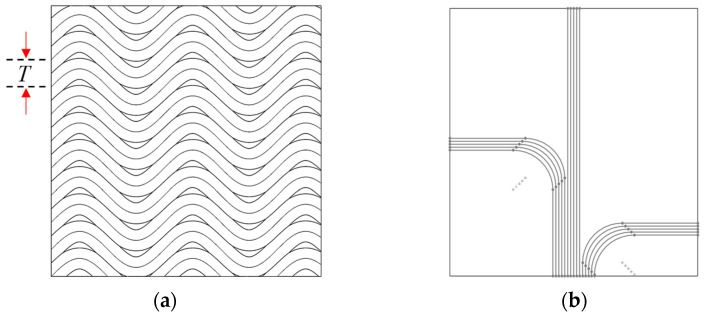
Sample patterns: (**a**) repeated offset pattern of the sinusoidal curve; (**b**) non-repeating sketch pattern driven by functional considerations.

This block is responsible for defining the parametric model of the curvilinear distributed V grooves. Curvilinear patterns require the use of the rotation tool center point (RTCP) function to be fabricated using SPDC. With the use of RTCP, these complex patterns can be further extended to complex workpiece geometries. Many different patterns can be generated by simply repeating the parametric curve defined by SFB 2. For instance, the unit sinusoidal V-groove generated with SFB 2 can be multiplied by a pattern, as shown in Figure 4a. To enable this, only one additional parameter, termed the step-over distance (*T*), is required. In addition to the examples presented in Figure 4, SFB 3 can be used to generate many other types of patterns whose complexity is generally limited solely by the possibility of defining and/or modeling them.

Once the areal pattern was generated according to the inputs provided to SFB 2 and SFB 3, the information was provided to the next processing modules of the workflow (MFB 2 and MFB 3), which are responsible for 3D modeling and generation of the cutting path/NC code, respectively.

## 4. CAD Module (MFB 2)

This module consists primarily of macros whose primary role is to automate the generation of a solid model to follow preset geometric parameters. The main advantage of macros is represented by their ability to rapidly model/remodel the geometry of the functional surface according to the selected parameters or those yielded by an iterative design optimization loop.

The input for the CAD module is defined in the parametric model as its output of the cross-section (*h_i_*, $\beta_{i}^{\mathrm{left}}$, $\beta_{i}^{\mathrm{right}}$, *i* = 1…*n*) alongside of the defined workpiece size (*LengthX*, *LengthY*, and *LengthZ*), the work coordinate system (WCS) origin, and the defined pattern. These inputs are combined to create 2D cross-sectional sketches, which can be found at the start of the curves ($t_{i}^{\mathrm{start}}$). The cross-sectional sketch follows the lofting curve (defined in a parametric or explicit form) until the final point $t_{i}^{\mathrm{end}}$. For the surface to be structured, the cross section is repeated for each groove (each characterized by its own 2D cross-sectional sketch), and then each individually chained/linked segment is extracted and placed into its own separate sketch. Two different options are provided in SBF 3. More specifically, if an explicit curve is provided, the underlying driving equation will be solely dependent on the *y* variable. By contrast, when the curve is provided in a parametric form, the two components of the position vector are to be provided as *x(t)* and *y(t)* dependencies. In case of a pattern, then only its type alongside with *h_i_*, $\beta_{i}^{\mathrm{left}}$, $\beta_{i}^{\mathrm{right}}$, *i* = 1…*n* have to be provided.

**Figure 5:**
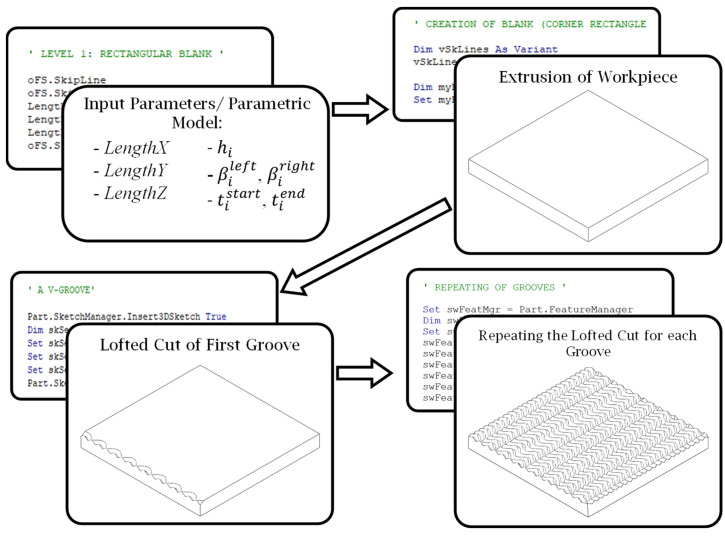
Flow diagram of CAD module.

Figure 5 presents the flow of information within the MFB 2. According to the flowchart, processing begins by generating a solid based on predefined dimensions. Subsequently, a 2D sketch of a sinusoidal shape was created on the top workpiece surface. Next, a second 2D sketch is drawn perpendicular to the endpoint of the first curve. These two sketches were subsequently combined using a ‘lofted cut’ to generate a groove in the workpiece. The groove created in this manner can be repeated as needed or modified individually to enable varying cross-sectional profiles. Thus, the entire top surface of the workpiece can be structured according to the design intent.

The resulting solid CAD model can then be used for advanced numerical simulations to evaluate various functional performances, such as drag reduction, self-cleaning, fouling resistance, or controlled aerodynamics/hydrodynamics. MFB 2 also enables the visualization of the functional structure before its fabrication via MFB 3 (CAM module) and can serve as a ‘digital master’ for quality inspection purposes.

**Figure 6:**
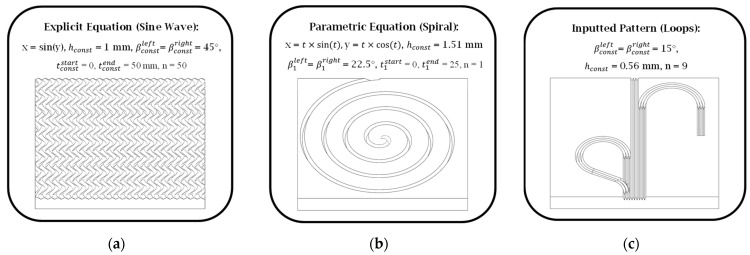
Curve modeling options: (**a**) explicit, (**b**) parametric, and (**c**) loops/pattern.

Figure 6 highlights the versatility and computational efficiency of the developed CAD macros for three representative input configurations. More specifically, Figure 6a demonstrates the use of an explicit analytical function (sinusoidal waveform) to define the geometry, whereas Figure 6b shows a parametric formulation exemplified by an Archimedean spiral.

In contrast, Figure 6c illustrates a pattern defined by a repeated cross-sectional profile. In this case, the only constraint imposed was that the input curve must be non-self-intersecting, because self-intersecting geometries are not supported by the underlying modeling kernel. This demonstration underscores the macro’s capability to accommodate a diverse range of functional surface designs. Furthermore, the macro can be reused with modified parameters in order to generate successive iterations of a given planar surface, thereby enabling efficient design refinements without relying on manual, tedious, and trial-and-error attempts.

## 5. CAM Module (MFB 3)

This module focuses on the output of the integrated CAD/CAM workflow: the NC code required for the fabrication of the functional surface to become a physical replica of the CAD model. The MFB 3 module receives input from the parametric modeling stage, supplemented by four additional secondary functional parameters. In contrast with commercial CAM software, a dedicated visualization engine is not incorporated within this module since geometric modeling is primarily handled by MFB 2. The only form of visualization provided by MFB3 is a Matlab-generated 3D representation of the tool path trajectory.

### 5.1. Tool Geometry (SFB 4)

**Figure 7:**
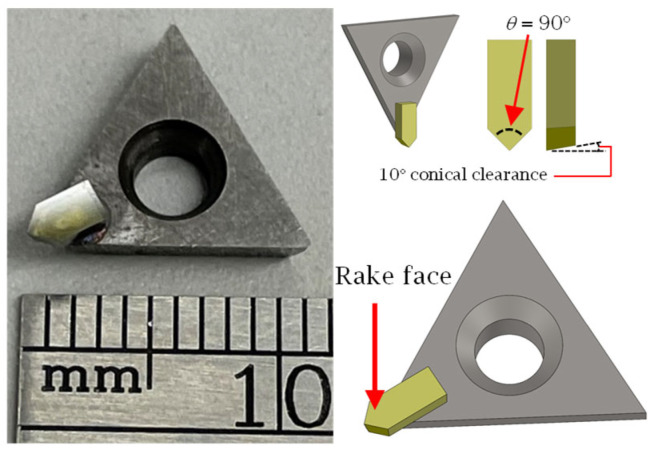
V-shaped monocrystalline single-point diamond cutting tool.

According to the proposed framework (Figure 1), the cutting tool geometry must be defined initially in SFB 2. Among the various micromachining strategies applicable to the fabrication of V-groove structures, micro-milling and single-point diamond cutting [17] are notable examples. The selection between them is generally dependent on the specifics of the application and the capabilities of the micromachining platform. Because the focus of the current study is the generation of functional surfaces consisting of symmetric curvilinear V-grooves, single-point diamond cutting (SPDC) remains the preferred manufacturing method primarily due to its precision and geometric fidelity. As shown in Figure 7, a V-shaped monocrystalline diamond insert mounted on a carbide holder represents the common tool/adaptor setup used for this purpose.

For toolpath generation, the CAM module requires a concise but complete set of geometric descriptors of the diamond insert, namely: (i) included (apex) angle (*θ*) that determines the V-groove facet orientation, (ii) rake angle required to compute the cutting edge normal, (iii) side and bottom clearance angles that prevent flank interference, (iv) cutting-edge (edge-hone) radius required to ensure adequate material removal, (v) tip height (tool-reference offset) defined as the distance from the insert datum to the cutting apex, which is required for RTCP positioning, and (vi) shank dimensions (width, thickness, overhang) that are required for collision and reachability checks within the post-processor. These six parameters are passed from SFB 4 to SFB 5–7, ensuring that the chordal deviation discretization, cutting strategy calculations, and RTCP transformations are performed in accordance with the tool geometry that is being used.

Given the emphasis on symmetric curvilinear V-grooves in this integrated approach, the most critical parameter of SFB 2 is represented by the included angle (*θ*) because the physical V-groove will materialize an inverse geometric replica of the cutting tool profile in the sense that the size of the included angle dictates the orientation of the two side/facet angles of the V-grooves. Some additional tool geometry parameters (rake and clearance angles) must also be set at this stage to ensure accurate process planning. Figure 7 presents two examples of monocrystalline diamond insert tools featuring a rake angle of 0°, with both the side and bottom clearance angles set at 10° on each symmetric cutting face. The inserts were precision-ground and lapped to achieve symmetric included angles of 90° and 30°. Once defined, these tool geometry parameters were propagated to SFB 5 for further integration into the manufacturing model.

### 5.2. Discretization of V-Groove Curve (SBF 5)

The primary function of SFB 5 is to discretize the geometric pattern generated by SFB 3 into a sequence of linear segments. This step is essential for enabling tool path generation in single-point diamond cutting (SPDC), where the alignment between the cutting trajectory and tool orientation is critical to ensure precise linear motion during fabrication.

**Figure 8:**
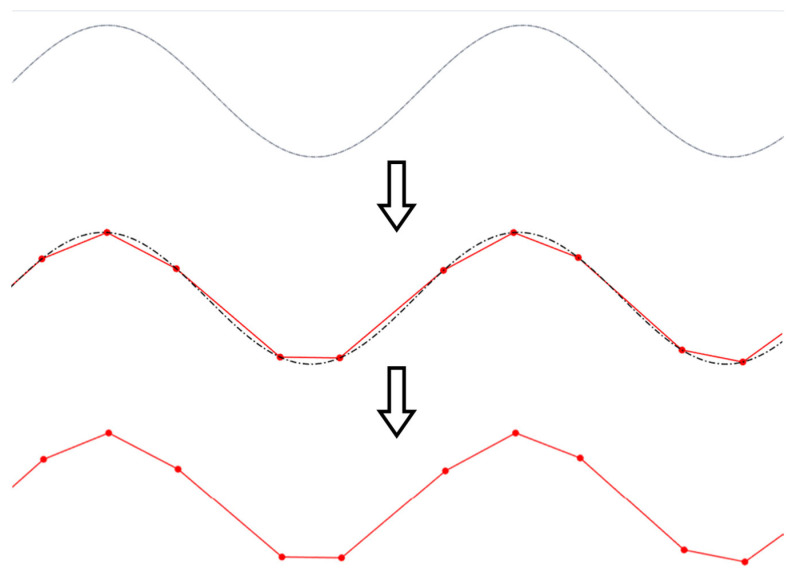
Discretization of a curve: from smooth to piecewise linear approximation based on chordal deviation.

Figure 8 illustrates the discretization of a (sinusoidal) curve into a series of straight segments. While discretization is a standard operation across various machining processes in which the numerical controller does not have higher-order interpolation capabilities, it is particularly crucial in SPDC due to the need for directional control of the tool motion.

**Figure 9:**
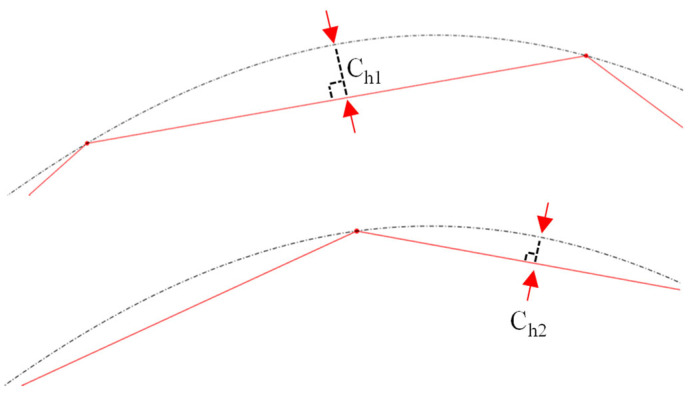
Curve approximation fidelity (C_h2_ < C_h1_).

Among the available discretization techniques, the chordal deviation method [23,24] was employed in this study due to its computational efficiency, simplicity, and geometric fidelity. As shown in Figure 9, the chordal deviation is defined as the maximum distance between the original curve and its linear piecewise/chordal approximation. Chordal deviation (C_h_), a user-defined input parameter, governs the resolution of the discretized paths. Smaller chordal deviation values translate into a higher number of linear segments and improved curve approximation fidelity (Figure 9).

To implement chordal deviation, the Newton-Raphson method is typically utilized to iteratively determine the location of the “next” point in the discretization set that obeys the prescribed C_h_ tolerance. After the completion of the discretization process, the generated tool path coordinates were passed to MFB 3 (CAM module) for NC code generation.

### 5.3. Cutting Strategy (SFB 6)

Achieving a unit V-groove with high surface quality (*S_a_* < 10 nm), burr-free edges, and precise form fidelity remains a significant technical challenge in ultra-precision machining. Secondary Functional Block 6 (SFB 6) addresses this by offering the possibility of multiple cutting strategies designed to balance machining efficiency with geometric and surface integrity. Numerous strategies have been proposed in the literature for the fabrication of functional surface structures, two of which have been integrated into the proposed framework: constant cutting area (CCA) and constant chip thickness (CCT) [17].

**Figure 10:**
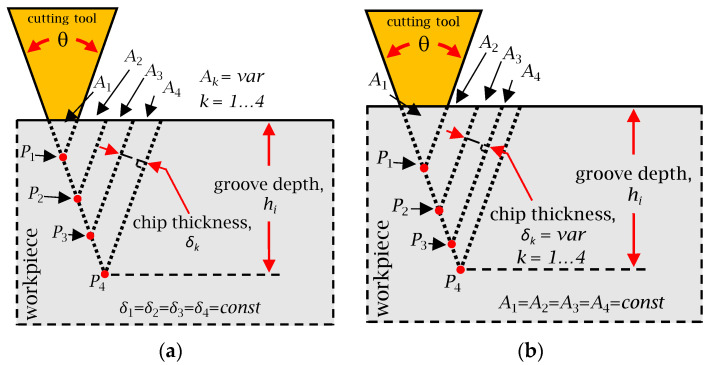
Single-flank cutting strategies: (**a**) constant chip thickness (CCT), and (**b**) constant cutting area (CCA).

As shown in Figure 10, both strategies rely on a single flank cutting approach, wherein both groove facets are formed simultaneously, but with an emphasis placed on the primary cutting flank. This cutting approach has advantages, such as reduced burr formation and a lower risk of damaging adjacent V-grooves. Nonetheless, alternative cutting strategies, including double-flank and alternating-flank cutting, could also be implemented in SFB 5 in order to potentially enhance productivity or improve surface finish, depending on the context of the application.

Figure 10a illustrates the first cutting strategy, constant chip thickness (CCT). This approach is widely regarded as one of the most practical for single-point diamond cutting (SPDC) due to its ease of implementation and minimal requirement for prior process planning [17]. As its name suggests, the defining characteristic of CCT is the maintenance of a uniform uncut chip thickness (*δ*_th_ = constant) throughout the cutting sequence. This was achieved by maintaining a constant depth of cut, with cutting points (P_1_, P_2_, P_3_, and P_4_) strategically defined along the groove profile. The final cutting point (P_4_) corresponds to the final groove depth, *h_i_*. Although the target chip thickness is preserved across the majority of toolpaths, the final increment may yield a smaller chip than prescribed if the remaining depth is less than *δ*_th_. Although the depth of cut remains constant, the cross-sectional area—and thus the volume of material removed—increases progressively with each pass. This results in a corresponding increase in the cutting forces, which can adversely affect the tool life, induce wear, and degrade the surface integrity. In severe cases, it may lead to tool fracture or chipping at the groove peak. Nonetheless, the primary advantages of the CCT strategy are its operational simplicity and high material removal rate, which contribute to a faster surface generation.

Figure 10b presents the second cutting strategy, the constant cutting area (CCA), which addresses several of the limitations associated with the CCT approach. While CCA offers improved control over cutting forces and tool integrity, it typically requires longer machining times than CCT. The core distinction between the two strategies lies in the parameter held constant: CCA maintains a uniform material removal area per cut (*A_i_* = constant). This implies that A_1_ = A_2_ = A_3_ = A_4_ (Figure 10b). The CCA approach translates into a uniform cutting volume and, therefore, stable cutting forces throughout the process. The preset incremental cutting area *A_i_* serves as a key input to the strategy and can be used to estimate the tool load per pass as follows: In instances where the final segment would result in a depth exceeding the predefined profile depth *h_i_*, the area of the final cut is automatically reduced to avoid overcutting. By maintaining constant cutting forces, the CCA strategy reduces the risk of tool wear, edge chipping, and deformation of the V-groove peaks. Although this method is more time-intensive, it is generally more favorable for extending the tool life and preserving the surface quality. This makes CCA suitable for machining scenarios in which surface quality and tool life are prioritized over simplicity and productivity, which are the core traits of the CCT strategy.

Depending on the specific requirements of the target functional structure, either of the two cutting strategies presented can be used within the developed CAD/CAM framework. Furthermore, alternative strategies, such as alternate-flank and double-flank cutting, may be employed to accommodate various geometric or process-specific constraints. The availability of multiple cutting strategies enables the optimization of machining parameters with respect to fabrication speed, surface quality, and tool longevity, depending on the intended application of the functional surface. Once a cutting strategy was chosen, the corresponding set of discretized toolpath points P*_j_*, *j* = 1…*p* can be forwarded to the subsequent functional block for NC code generation.

### 5.4. Cutting Process Plan (SFB 7)

The final processing stage of the CAM module involves the actual fabrication of the surface as a series of V-grooves. This is handled by Secondary Functional Block 7 (SFB 7), labeled as “Process Plan” in Figure 1. This block governs the selection of the machining strategy used to generate a complete functional surface. In the context of single-point diamond cutting (SPDC), the post-processing of the tool path requires a continuous adjustment of the surface orientation to ensure that the vector normal to the primary rake face of the cutting tool remains confined within the vertical plane that contains the feed direction associated with each discretized segment (as generated in SFB 5). Depending on the configuration of the micromachining system, achieving the desired orientation along the groove path typically requires rotational movements about one of the principal axes of the machine tool.

**Figure 11:**
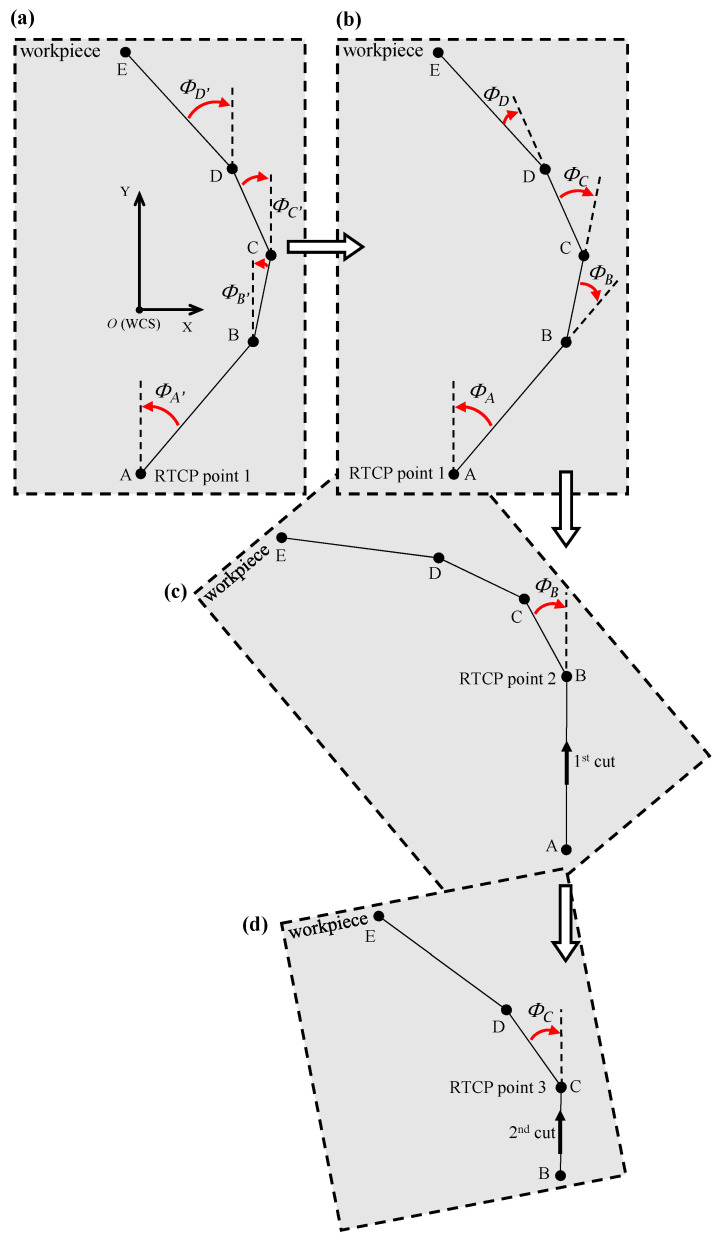
Successive *Z*-axis rotations to ensure the alignment between the tool path segments and cutting axis: (**a**) discretized tool path trajectory; (**b**) rotational angles; (**c**) first cut; (**d**) second cut.

In the example presented in Figure 11, the direction of cutting is aligned with the *Y*-axis of the machine tool, which likely means that a rotation about the *Z*-axis is required to reorient the workpiece in the proper direction. The underlying assumption here is that the machine tool has a kinematic structure, as outlined in [25], namely, a rotary table configuration capable of *B* and *C* rotations.

The angular increments were calculated as follows:

(1) $$\phi_{A\prime }=\tan^{-1}(B_{X}/B_{Y}),$$

and

(2) $$\phi_{B}=\phi_{A\prime }-\phi_{B\prime }.$$

Equation (1) computes the absolute angular orientation of each discretized point with respect to the Y-axis based on its original pre-rotation coordinates. These absolute angles were subsequently converted to incremental angles using Equation (2) since the NC code generated for the SPDC process operates in an incremental post-rotation mode. Figure 11c,d illustrate the complete reorientation of the tool path following each rotational adjustment. Rotation is performed about the *C*-axis, corresponding to the physical rotation of the workpiece, prior to initiating each cut. The reoriented tool path for the first cut is depicted in Figure 11c, and Figure 11d shows the updated path for the subsequent cut after another incremental rotation. This process is repeated iteratively for each segment along the tool path until the full surface structure is machined.

**Figure 12:**
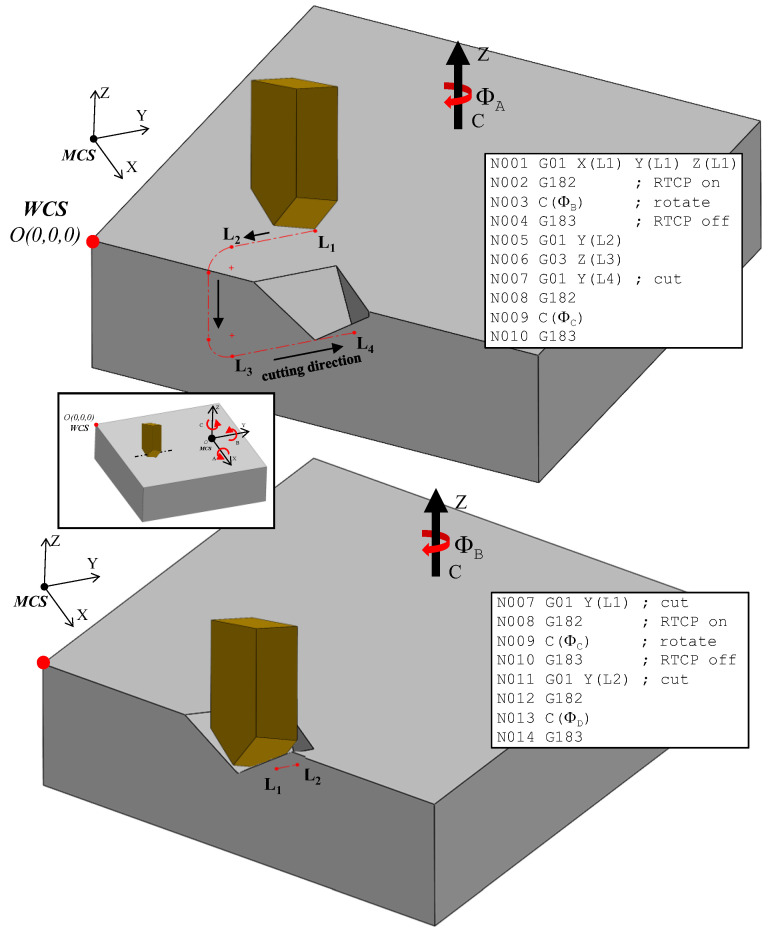
Workpiece rotation during curvilinear curve cutting.

A critical challenge in 5-axis machining arises from the transformation of surface points relative to the global coordinate system of the machine during each rotation. As illustrated in Figure 12, the workpiece was rotated within the machine coordinate frame, which is separately shown in the inset box using the *Z*-axis indicator. If the point of rotation, that is, the transition point from one cut to the next, does not coincide with the center of the rotary axis, the start point for each subsequent segment must be recalculated following each incremental rotation. This redefinition ensures the alignment between the tool path and the updated workpiece orientation, thus preserving the dimensional accuracy and trajectory consistency throughout the entire machining process.

Two primary methods are currently available for redefining tool or point positions following rotational movements available on multi-axis machining systems. The first approach involves implementing a user-defined or system-specific transformation matrix within the CNC post-processor. This method can be adapted to a broad range of CNC controllers, including those lacking built-in rotation compensation capabilities. The second technique uses the rotation tool center point (RTCP) functionality, a manufacturer-provided feature embedded within some advanced controllers. Although the RTCP simplifies tool orientation management during rotational movements, it is available only on select high-end commercial controllers.

As outlined in the past [26], the transformation matrix-based approach is highly customizable but demands an in-depth understanding of spatial coordinate transformations/inverse kinematics, particularly for five-axis machines. The general transformation matrix must be generated according to the specifics of the machine tool kinematics, which in turn means that each matrix is only applicable to the machine tool configuration for which it was determined. Once all its components are known, the matrix can be integrated within the post-processor, thereby enabling the recalculation of the rotational pivot point as well as the entire tool path trajectory in the machine coordinate system (MCS), which is the only one that is a priori known to the numerical controller. This recalculated trajectory is passed directly to the CNC controller, eliminating the need for external computation during machining. Although not difficult, this approach requires advanced knowledge of kinematics and is rather tedious since the general transformation matrix is only applicable to the machine tool for which it was determined.

In contrast, the RTCP approach dynamically maintains the cutting tool tip at a fixed location relative to the workpiece coordinate system (WCS) while allowing the workpiece to rotate as needed about the *Z*, *X*, or *Y*-axes. The RTCP relies on an inbuilt transformation matrix and automatically adjusts all tool position coordinates in real time as the workpiece rotates. When RTCP is activated, programming is simplified since all tool motions remain expressed in the WCS without the need to be converted to and from the MCS. The RTCP approach requires precise positioning of the WCS origin within the MCS and accurate alignment between the WCS and MCS axes; however, unlike the previous approach, it is more straightforward and does not require external-to-controller calculations. Evidently, all these advantages come at the expense of a higher cost for the controllers.

**Figure 13:**
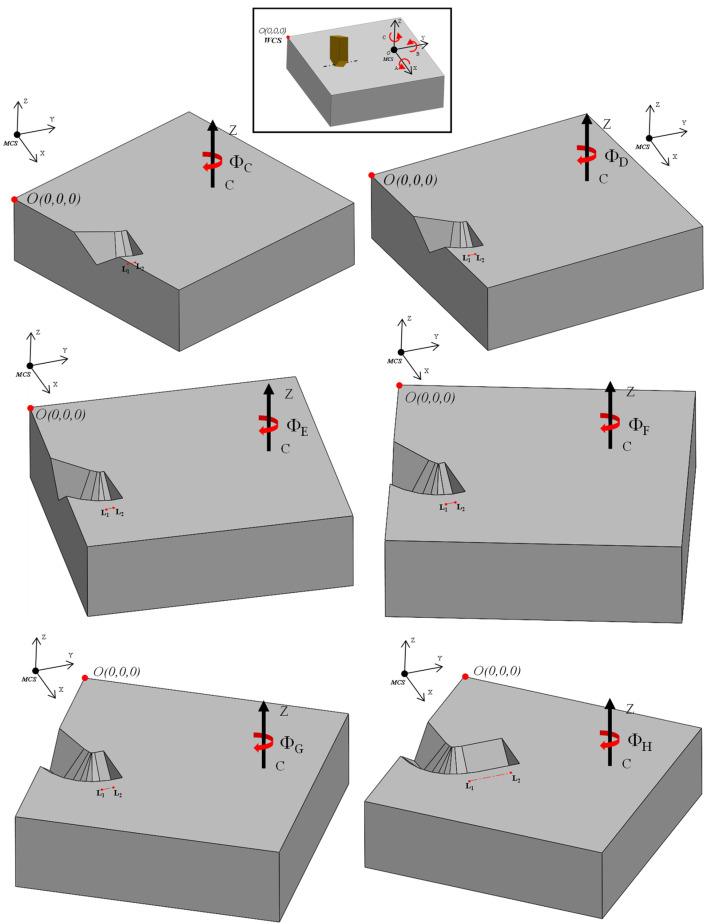
Overview of the curvilinear V-groove cutting process.

Figure 12 and Figure 13 illustrate the implementation of discretization in the fabrication of curvilinear V-groove functional structures. Both figures continue the example of the sinusoidal curve shown above in the sense that they are built on the information detailed about SFB 2, 3, and 6. To initiate the tool path trajectory for this type of structure, the cutting tool must first be positioned above the initial groove segment at a predefined height above the workpiece (L_1_ in Figure 12). Once in position, the RTCP function is activated, and the workpiece is rotated about the *C*-axis by an angle of *Φ*_A_. The RTCP function is then deactivated, aligning the direction of the first cut with the machine’s Y-axis. The tool is subsequently moved to point L_2_ and then lowered to L_3_ via a circular interpolation move rather than a linear descent. This lead-in motion improves tool stability, reduces the likelihood of motion-induced vibrations, and facilitates chip evacuation, as discussed in [17]. The location of L_2_ and L_3_ is determined based on the start parameter of the sinusoidal curve, $t_{i}^{start}$ that is dependent on the input geometry and may lie within the workpiece boundary. Point L_3_ is aligned to the cutting depth corresponding to the first point P_1_, which is defined by the selected cutting strategy. The initial cutting operation then proceeds from L_3_ to L_4_, where the endpoint L_4_ is determined by the chordal deviation. This implies that the segment length varies according to the local curvature of the curve being discretized/traced.

After the first cut is completed, the tool remains at point L_4_ to prepare for machining the next segment. The second part of Figure 12 depicts the process for the subsequent segment, for which the toolpath sequence is repeated with reinitialized labeling for clarity. The RTCP function is reactivated, and the workpiece is rotated by the next angle *Φ*_B_, aligning the new segment with the *Y*-axis for the second cut. The CNC code structure corresponding to this iterative process is also shown in Figure 12, highlighting the cyclic nature of the commands. Notably, the location of L_1_ in the new sequence corresponds to L_2_ from the previous cut, while the updated L*_2_* coincides with the former L_1_ to maintain toolpath continuity. The toolpath and cutting sequence shown in Figure 13 correspond to the second iteration, demonstrating the repetitive yet synchronized nature of the multi-segment groove fabrication.

Figure 13 illustrates the benefits of employing the chordal deviation discretization approach in generating smooth, optimized tool paths for curvilinear V-groove cutting. This motion is iteratively executed along the curve until reaching its terminal point ($t_{i}^{end}$), thereby completing the groove at the target depth corresponding to point P_1_. After that, the groove can be progressively deepened by means of repeated passes until the target depth is reached. Once the groove is completed, subsequent grooves can be machined by laterally moving the tool in the X-direction using a step-over distance that is consistent with the spacing of the periodic pattern to be fabricated. This cutting sequence can then be repeated for each groove, such that the entire workpiece can be structured according to the intended design of the functional surface. Moreover, grooves can be machined sequentially or in an alternating/arbitrary pattern, depending on the selected process strategy. The chosen groove arrangement can significantly influence the quality and performance of the final functional surface. For instance, the sequence of cuts may be optimized to minimize the cumulative deformation of adjacent riblets, reduce burr formation, enhance surface quality, and fulfill performance criteria related to aerodynamics, hydrodynamics, flow control, or optical applications.

Once the process plan for fabrication is completed, by integrating the outputs from SFB 4, SFB 5, SFB 6, and MFB 1, numerical control (NC) code can be automatically generated using the CAM module (MFB 3). The NC code encompasses the full machining logic presented in Figure 12 and Figure 13 and includes all discretized toolpath points (SFB 5), cutting strategy decisions (SFB 6), and tool orientation instructions (SFB 7). When operating within the WCS, the use of the RTCP function greatly simplifies the NC code generation process. In RTCP-enabled systems, tool orientation and spatial transformations are handled natively by the CNC controller, eliminating the need for manual coordinate recalculation by an external programmer. However, if the controller lacks the RTCP functionality, a system-specific transformation matrix must be developed and integrated into the post-processor (MFB 3) in order to accurately map the tool path after rotational movements.

By ensuring its compatibility with both RTCP-capable and conventional CNC systems, the integrated CAD/CAM approach offers a flexible and robust solution for the automated fabrication of curvilinear V-groove-based functional surfaces. Once MFB 3 is completed, the developed framework is practically ready for final implementation and testing.

## 6. Framework Validation

Two functional surfaces were fabricated to evaluate the applicability and functionality of the integrated CAD/CAM approach. The first sample featured a periodic sinusoidal V-groove pattern designed to demonstrate the system’s capability to machine complex geometries in aluminum while maintaining a high surface quality. The second sample was fabricated to illustrate the versatility of the approach in producing structures intended for functional applications, in this case, to enable controlled open micro-capillary flow. The two examples presented next were meant to validate the effectiveness of the integrated framework with respect to precision machining and functional surface engineering.

### 6.1. Case Study 1: Sinusoidal Pattern Fabrication

**Figure 14:**
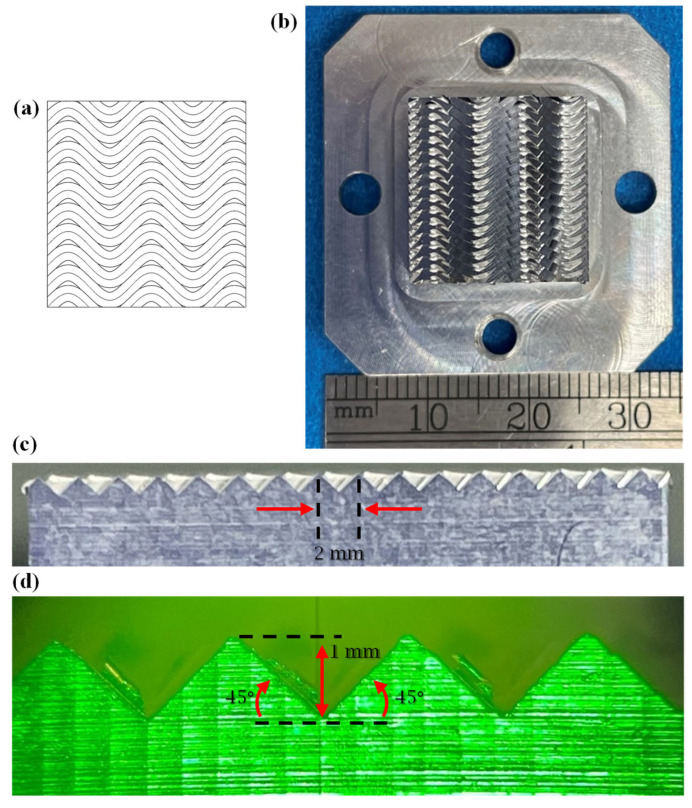
Functional sinusoidal pattern fabricated on a flat sample: (**a**) CAD model, (**b**) physical prototype, (**c**) cross-sectional overview, and (**d**) cross-sectional detail.

To demonstrate the applicability of the proposed framework, a functional surface featuring a sinusoidal V-groove pattern was fabricated from an aluminum workpiece with dimensions of 17 mm × 17 mm × 10 mm (length × width × thickness), as shown in Figure 14. Each groove was machined to a constant depth of *h*_const_ = 1 mm and facet angles of $\beta_{\mathrm{const}}^{\mathrm{left}}=\beta_{\mathrm{const}}^{\mathrm{right}}=45{}^{\circ}$. The groove extended across the entire workpiece length (from $t_{i}^{\mathrm{start}}$ to $t_{i}^{\mathrm{end}}$), resulting in a full curve profile within each groove. The parametric expression of the sine curve was provided to the CAD macro, together with a spatial period of 1 mm. This yielded 17 grooves across the surface. The resulting digital model (Figure 14a) closely replicates its physical counterpart (Figure 14b).

To fabricate the physical sample, a single-point diamond tool characterized by a 90° included angle and 10° clearance angle (Figure 7) was used to trace the sinusoidal V-grooves discretized with a 0.01 mm chordal deviation. The CCT strategy (Figure 10a) was employed for groove fabrication primarily because of its simplicity. The chip thickness was set to 10 μm, and the cutting speed was set to 1800 mm/min. The RTCP function was activated, and the grooves were cut by employing a first pass across all of them, followed by repeated incremental passes until the final depth was reached.

**Figure 15:**
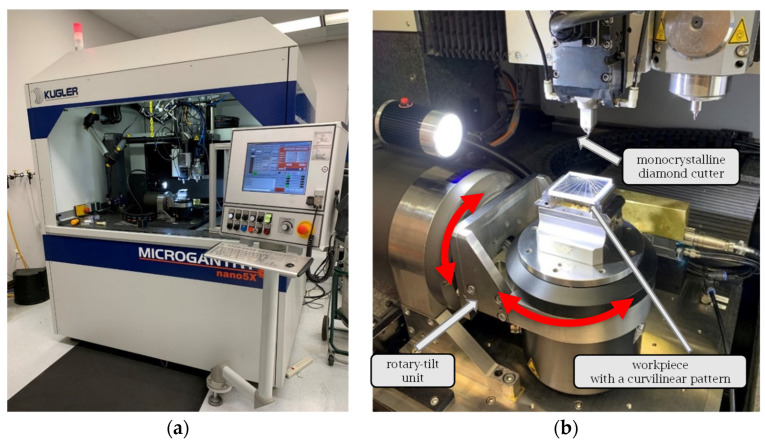
Fabrication setup: (**a**) overview of the five-axis micromachining center, (**b**) cutting setup.

The NC code was generated using a custom-built post-processor written in MATLAB. Prior to the actual cutting, the generated NC code was verified in Vericut to ensure its collision-free operation and trajectory correctness. After validation, the NC code was executed on a Microgantry Nano 5x multi-axis micromachining center (Figure 15a). This precision system features a positioning accuracy of less than ±1 μm and a maximum feed rate of 10,000 mm/min. More details of the fabrication setup are presented in Figure 15b. As shown, the SPDC process was implemented with the assistance of an *AC* rotary table mounted along the y-axis. The completed prototype is presented in Figure 14b, with the groove cross-section shown in Figure 14c and an enlarged annotated view in Figure 14d, detailing the key geometric parameters and periodicity of the fabricated structure.

### 6.2. Case Study 2: Capillary Flow Functionality

**Figure 16:**
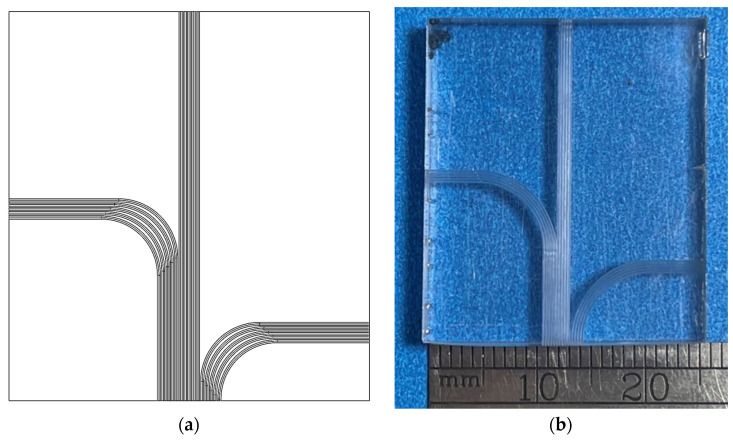
Functional curvilinear pattern fabricated on a flat sample: (**a**) digital model; (**b**) physical sample.

To demonstrate the functional versatility enabled by the proposed CAD/CAM approach, a microstructured surface was fabricated to test the open vertical capillary flow (Figure 16a). The sample was machined from an acrylic substrate with dimensions of 25 mm × 25 mm × 5 mm (length × width × thickness). Each groove had a constant depth of *h*_const_ = 0.4 mm and symmetrical flank angles $\beta_{\mathrm{const}}^{\mathrm{left}}=\beta_{\mathrm{const}}^{\mathrm{right}}=15{}^{\circ}$. The groove trajectory was manually defined and input into the CAD macro. The resulting digital model (Figure 16a) closely matched the fabricated physical sample shown in Figure 16b.

The intended toolpath was discretized using a chordal deviation of 0.01 mm to ensure high geometric fidelity. A single-point diamond cutting tool with an included angle of 30° and a clearance angle of 10° was used to machine the structure. The constant cutting area (CCA) strategy (Figure 10a) was used to machine the V grooves. The cutting strategy was characterized by a chip thickness of 10 μm, a cutting speed of 1800 mm/min, and an active RTCP function.

**Figure 17:**
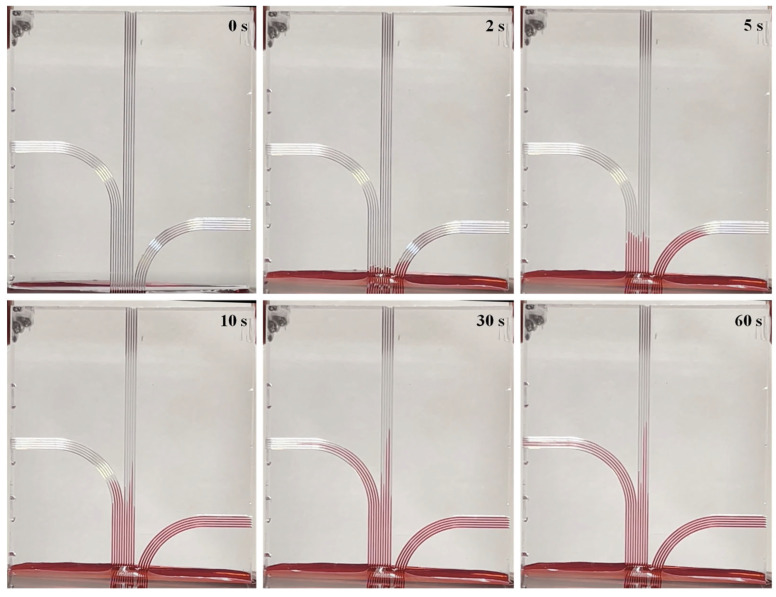
Rise of the liquid through the curvilinear V-grooves under the action of capillary forces (timestamp in seconds in the top-right corner of the images).

The surface shown in Figure 16b was fabricated to test the applicability of the integrated CAD/CAM approach in a specific functional application, namely, open capillary flow (Figure 17).

For this purpose, the microfluidic experiment was initiated by gradually raising a crank stand supporting a water reservoir until the fluid surface contacted the lower edge of the grooved structure (Figure 17, 0 s). Upon contact, capillary forces drove the fluid upward through the v-grooves. This phenomenon has been widely characterized in prior studies [27,28,29]. Rapid fluid propagation was observed in the curved regions of the groove network, suggesting that curvature may enhance the flow dynamics. This trait can be exploited in future studies. Furthermore, the height of the liquid increased progressively with time, confirming the grooves’ ability to promote continuous capillary rise. Taken together, these observations support the effectiveness of the proposed framework in producing microstructured surfaces with functional capabilities relevant to flow manipulation and capillary-driven transport.

## 7. Conclusions

This study presents a comprehensive integrated CAD/CAM framework for the parametric design and fabrication of curvilinear V-groove-based functional surfaces. The methodology is structured around three primary function blocks (MFBs): parametric model (MFB 1), CAD module (MFB 2), and CAM module (MFB 3). MFBs are assisted by seven secondary function blocks (SFBs) responsible for the essential inputs related to workpiece geometry, unit groove design, curvilinear pattern generation, curve discretization, cutting tool geometry, machining strategy, and cutting process planning. The proposed approach enables the automated generation of a parametric model of the functional surface, a corresponding solid model, and numerical control (NC) code required for its fabrication via single-point diamond cutting (SPDC). The framework’s functionality was validated through the successful fabrication of several structured prototypes using an advanced micromachining platform capable of rotation tool center point (RTCP) functionality. One of the fabricated samples demonstrated the effectiveness of curvilinear V-grooves in facilitating open vertical capillary flow, thereby illustrating the applicability of the developed framework in microfluidic surface engineering. Nonetheless, the framework’s versatility can be easily extended to a wide range of functional applications for which V-grooved microstructures represent a viable option.

Although the two case studies presented were demonstrated in a research-oriented fabrication setup, the developed software architecture is intentionally platform agnostic. More specifically, the post-processor exports a standard ISO G-code that is compatible with mainstream five-axis machines equipped with RTCP capabilities. For large-area surfaces, the chordal deviation routine can be invoked in a tiled/batch mode, while the MATLAB back end supports multi-threading to preserve reasonable processing times. Therefore, porting the developed CAD macros to commercial environments would be straightforward via their open APIs, even though it is expected that the curve parameter and tool-library calls will require a certain degree of editing and/or customization. Nevertheless, industrial deployment will also require (i) adaptive edge-radius compensation tables to manage tool wear over long runs, (ii) rigid-body damping or active vibration control on larger machines, and (iii) an optional link to a manufacturing execution system (MES) for closed-loop parameter updates. With all these adaptations in place, it is anticipated that the proposed framework could scale from millimeter-scale laboratory specimens to wafer- or panel-scale production without any major alterations to the core algorithms.

Future extensions of this work will present additional application examples by incorporating both geometric and physical evaluations of V-groove-based surfaces. Immediate application areas include the fabrication of functional surfaces for drag reduction, fouling resistance, light guiding, and open microfluidics. Future development efforts will focus on extending the framework into a flexible and fully integrated CAD/CAM solution capable of generating design and fabrication strategies for complex micro- and nano-scale surface features to be manufactured on a broad range of freeform geometries.

## Data Availability

The original contributions presented in this study are included in the article. Further inquiries can be directed to the corresponding authors.
